# Identification of Effective Coefficients of Thermal Contraction of Structural States in Cast Bronze by Solving Inverse Problems of Thermodeformation Modeling

**DOI:** 10.3390/ma19214622

**Published:** 2026-10-30

**Authors:** Tetiana Kimstach, Hennadii Shvachych, Kostiantyn Uzlov, Oleksandr Bilyi, Serhii Repiakh, Serhii Semenov, Magdalena Krupska-Klimczak

**Affiliations:** 1 Department of Material Science and Heat Treatment of Metals, Ukrainian State University of Science and Technologies, 49010 Dnipro, Ukraine; 1375tatyana@gmail.com (T.K.); konst.uzlov@gmail.com (K.U.); 2 Department of Problems of Thermomechanical Processing of Structural Steels, Iron and Steel Institute of Z. I. Nekrasov of National Academy of Sciences of Ukraine, 49107 Dnipro, Ukraine; 3 Department of Software Engineering, Dnipro University of Technology, 49005 Dnipro, Ukraine; 4 Department of Casting Production, Ukrainian State University of Science and Technologies, 49010 Dnipro, Ukraine; baplitvo@gmail.com (O.B.); 123rs@ua.fm (S.R.); 5 Institute of Security and Computer Sciences, University of the National Education Commission, ul. Podchorazych 2, 30-084 Krakow, Poland; magdalena.krupska-klimczak@uken.krakow.pl

**Keywords:** bronze, free linear shrinkage, thermal contraction, structural states, inverse identification, phase transformations, cascade modeling, IA-CLTC

## Abstract

The temperature-dependent formation of free linear shrinkage in cast CuAl7Si2Sn1.5Mn0.3 aluminum bronze was investigated using a cascade inverse-identification approach. Experimental data for 17 bronze compositions (*K*ᵣ = 0.41–0.85), including differential thermal analysis, quantitative metallography, and free-linear-shrinkage measurements, were used to reconstruct three genetic structure-formation pathways. Unlike conventional phase-averaging approaches, the proposed methodology identifies effective integral-average coefficients of linear thermal contraction (IA-CLTCs) of successive structural states directly from integral shrinkage data without requiring independently prescribed thermophysical properties of individual phases. The identified IA-CLTC sequences were 20.10 → 15.44 → 18.99 × 10^−6^ K^−1^ for pathway I, 21.88 → 29.40 → 11.57 × 10^−6^ K^−1^ for pathway II, and 25.90 → 36.65 → approximately 11.67 × 10^−6^ K^−1^ for pathway III. The first coefficient of pathway I was adopted as a fixed reference value. The model reproduced the experimental shrinkage with R^2^ = 0.998 and RMSE = 0.00562 percentage points. Conditional cascade leave-one-composition-out cross-validation yielded RMSE = 0.00995 percentage points, compared with 0.00915 percentage points for a simple linear-regression benchmark. Near the boundary between pathways II and III, the reconstructed thermal and structural-transformation contributions exhibited nearly compensating changes, although their quantitative separation remained model-dependent. Numerical sensitivity analysis revealed strong parameter correlation and limited practical identifiability for pathway III. The proposed approach therefore provides a framework for reconstructing effective temperature–deformation characteristics of successive structural states, while independent experimental validation remains necessary for their broader predictive and physical interpretation.

## 1. Introduction

The dimensional and geometric accuracy of castings is substantially influenced by changes in their linear dimensions during solidification and subsequent cooling. Inaccurate prediction of free linear shrinkage complicates the determination of pattern dimensions and machining allowances, increases material consumption and machining requirements, and may contribute to residual stresses, distortion, cracking, and dimensional rejection of cast components [1,2,3,4]. Therefore, quantitative characterization of the temperature-dependent deformation behavior of casting alloys remains an important problem in foundry engineering.

Free linear shrinkage is an integral characteristic reflecting the combined effects of thermal contraction and structural evolution during cooling. For structurally stable single-phase materials, dimensional changes can be described by integrating the temperature-dependent coefficient of linear thermal expansion (CLTE) over the corresponding temperature interval [5,6,7,8,9]. However, this approach becomes substantially more complicated for multiphase casting alloys, in which phase transformations, changes in the proportions and morphology of structural constituents, and their mechanical interactions influence the resulting macroscopic deformation [10,11,12,13,14,15,16,17,18]. Consequently, the final shrinkage of such alloys cannot generally be represented as a simple sum of the unconstrained thermal strains of individual phases.

This problem is particularly relevant to multicomponent aluminum bronzes, whose structural states develop through successive transformations during solidification and cooling. Changes in chemical composition, even within the nominal range of a single alloy grade, can modify the characteristic temperatures of phase transformations, the sequence of structural-state formation, and the quantitative proportions of structural constituents [10,11,12,13,14]. Moreover, structural constituents of identical crystallographic nature may have different origins and formation histories. Therefore, similar final phase compositions do not necessarily imply identical temperature–deformation behavior during cooling.

Conventional approaches to calculating the effective thermal expansion of heterogeneous materials are generally based on homogenization of the thermophysical and mechanical properties of individual phases, taking into account their volume fractions and mutual mechanical constraints [5,6,7,8,9]. Although these approaches provide a physically meaningful description of multiphase deformation, their application to structurally evolving cast alloys requires prior knowledge of the temperature-dependent properties, phase proportions, and mechanical interactions of the constituent phases. Such information is often incomplete or unavailable for multicomponent bronzes undergoing successive structural-phase transformations.

An alternative approach is to formulate the problem inversely. Instead of calculating macroscopic shrinkage from prescribed phase properties, experimentally measured free linear shrinkage can be used, together with characteristic transformation temperatures and quantitative structural parameters, to identify effective temperature–deformation characteristics of the structural states formed during cooling. This formulation is consistent with established inverse-problem methodologies used for identifying material parameters that cannot be measured directly [19,20,21,22,23,24]. However, the inverse identification of internal deformation characteristics requires careful consideration of mathematical identifiability, parameter correlations, sensitivity to experimental data, and the physical interpretation of the resulting coefficients.

For the Cu–Al–Si–Mn–Sn bronze system, previous studies have established relationships between chemical composition, the amount of secondary structural constituents, mechanical properties, and free linear shrinkage [25,26,27,28,29]. Nevertheless, correlations between the final microstructure and macroscopic shrinkage do not independently reveal the contributions of successive structural states and temperature intervals to the accumulated deformation. A more detailed interpretation requires joint consideration of experimentally determined transformation temperatures, quantitative characteristics of the final structure, and the integral deformation response of the alloy.

To describe these relationships, the present study employs effective integral-average coefficients of linear thermal contraction (IA-CLTCs). Unlike conventional CLTE values assigned to individual crystallographic phases, these coefficients characterize the integral temperature–deformation response of reconstructed structural states over specified cooling intervals. For multiphase states, an IA-CLTC may indirectly reflect the combined influence of constituent proportions, morphology, spatial distribution, and internal mechanical interactions. Accordingly, the identified coefficients are treated as effective parameters of the adopted temperature–phase model rather than universal thermophysical constants of individual phases or the alloy as a whole.

The principal methodological distinction of the proposed approach lies in the physical organization of the inverse problem. The objects of identification are not isolated crystallographic phases but genetically successive structural states reconstructed from experimentally determined characteristic transformation temperatures, quantitative metallographic observations, and physically motivated phase-evolution schemes. The effective IA-CLTCs of these states and the structural-transformation coefficients are determined from integral free-linear-shrinkage measurements through a cascade identification procedure. Parameters obtained at preceding stages are subsequently used to constrain the identification of genetically related structural states. Thus, the methodological contribution consists of combining temperature–phase reconstruction with sequential inverse identification of effective deformation characteristics, rather than in introducing a new least-squares algorithm.

At the same time, the reconstruction of genetic structure-formation pathways from thermal analysis, final-state metallography, and phase-equilibrium considerations does not establish a unique sequence of intermediate crystallographic phases. Similarly, the decomposition of integral free linear shrinkage into thermal and structural-transformation contributions is conditional on the adopted structural schemes, functional parameterization, and assumptions used to close the inverse problem. Consequently, accurate reproduction of the experimental shrinkage data must be distinguished from the physical uniqueness and practical identifiability of the internal model parameters.

## 2. Literature Review

In foundry practice, the free linear shrinkage of an alloy is traditionally used as a technological parameter for determining pattern dimensions and predicting the final dimensions of castings [1,2,3,4]. However, the actual dimensional change in a casting depends not only on the temperature and properties of the alloy but also on its mechanical interaction with the mold [30,31,32,33,34,35,36]. A distinction between free and restrained shrinkage is therefore essential: the latter includes an additional deformation contribution from the mechanical resistance of the mold, whereas free shrinkage more directly characterizes the thermal and structural response of the material itself.

At the same time, using only the final free-shrinkage value has a substantial limitation: as an integral characteristic, it does not itself reveal the temperature intervals or successive structural states responsible for the final casting strain. The conventional technological approach is therefore sufficient for estimating the final dimensional change but insufficient for analyzing its temperature–structure origin.

The CLTE and free linear shrinkage share a thermal origin but are not identical characteristics. The CLTE describes the local change in linear dimension with temperature, whereas final linear shrinkage is the integral result of deformation over the entire cooling interval. For a structurally stable single-phase material, these quantities may be related by integrating the temperature-dependent CLTE. For a multiphase cast alloy, this approach is limited because the structural state of the material itself changes with temperature.

Experimental data for Cu–Al alloys confirm that the CLTE depends on temperature, Al content, and structural state [37,38]. In multiphase aluminum bronzes, phase transformations and mechanical interaction between structural constituents are also important [5,6,7,8,9,10,11,12,13,14,15,16,17,18,39]. Directly assigning reference or experimentally determined CLTE values of individual phases to successive multiphase states of a cast bronze is therefore methodologically insufficient. Such values may serve as physical benchmarks, but they cannot uniquely describe the integral temperature–deformation response of a structural state that evolves during cooling.

Models that account for constituent volume fractions, elastic properties, and mechanical constraint have been proposed to estimate the effective thermal expansion of heterogeneous materials [5,6,7,8,9]. The Turner and Schapery models are among the fundamental approaches of this type [5,6]. Their clear advantage is that they account for mechanical interaction between the constituents. However, their critical limitation for the present class of cast bronzes is the need for a priori knowledge of the temperature-dependent characteristics, elastic properties, and proportions of all constituents in each structural state. Such comprehensive information is essentially unavailable for a multicomponent alloy whose structure evolves successively during cooling.

A further limitation of classical phase averaging is that it does not in itself account for the structural-phase history of the material. In aluminum bronzes, changes in chemical composition and cooling conditions may affect not only the amounts of the phases but also their formation sequence [10,11,12,13,14,15,16,17,18,39]. Constituents of the same crystallographic nature may therefore have different origins, and a formally identical phase composition may exhibit a different temperature–deformation response. A description based only on final phase volume fractions cannot capture the genetic differences among structural states. Under these conditions, the appropriate object of temperature–deformation analysis is not only an individual phase but also the structural state formed at a particular stage of the temperature–phase evolution of the cast alloy during cooling.

In addition to thermal contraction, the final strain may be affected by structural-phase transformations accompanied by changes in specific volume and in the proportions of structural constituents. Part of the associated deformation effect may relax at high temperatures, whereas the unrelaxed part contributes to the final change in linear dimension. Approaches that describe shrinkage exclusively through thermal contraction cannot account separately for this residual structural-transformation contribution. An integral description should therefore distinguish between the thermal and residual structural-transformation contributions without attributing the latter to a specific micromechanism of a phase transformation in the absence of independent experimental confirmation.

A direct formulation for such an alloy would require the temperature–deformation characteristics of all successive structural states and the deformation effects of the transitions between them. No such complete dataset is currently available for a real multicomponent cast bronze. By contrast, the final free linear shrinkage, the temperatures of characteristic thermal effects, and quantitative parameters of the final structure can be measured experimentally. This makes it possible to formulate the problem inversely, that is, to determine effective characteristics of the structural states from integral experimental information.

Inverse problems are widely used in thermophysics to identify parameters that are difficult to determine directly [19,20,21,22,23,24,40,41,42,43]. However, this approach also has fundamental limitations: inverse problems may be ill-conditioned, contain correlated parameters, and be sensitive to errors in the input data [40,41,42,43,44,45]. Accurate reproduction of integral shrinkage alone is therefore not sufficient evidence that the identified internal parameters are physically unique. Such models require examination of system rank and conditioning, analysis of sensitivity to the composition of the experimental sample, and statistical assessment of the stability of the resulting characteristics.

For the investigated Cu–Al–Si–Mn–Sn system, differential thermal analysis, quantitative metallography, and free-linear-shrinkage measurements provide the experimental basis for reconstructing structural-phase evolution. The characteristic temperatures and the sequence of possible structural states were also interpreted with reference to the equilibrium Cu–Al phase diagram and established phase-transformation behavior in aluminum bronzes [10,11,12,13,14,15,16,17,18,26,39]. The genetic pathways used in the mathematical model should therefore be regarded as structural-phase schemes reconstructed from a set of independent experimental and thermodynamic indicators, rather than as direct observations of every intermediate phase state.

Thus, the literature extensively addresses separate aspects of the problem: dimensional changes and mechanical interaction between a casting and its mold [30,31,32,33,34,35,36], structure formation in aluminum bronzes [10,11,12,13,14,15,16,17,18,25,26,27,28,29,39], thermal expansion of Cu and Cu–Al alloys [37,38], models of effective thermal expansion in heterogeneous materials [5,6,7,8,9], and methods for solving inverse heat-transfer problems [19,20,21,22,23,24,40,41,42,43]. Existing approaches, however, generally address only individual parts of the problem and do not simultaneously describe the genetic sequence of structural states, their temperature–deformation characteristics, the structural-transformation contribution, and the stability of parameter identification.

The methodological gap addressed here is the formulation of an inverse description in which the effective response of successive reconstructed structural states, rather than a complete set of intrinsic phase properties, is identified from integral shrinkage and complementary thermal and structural data. This extends the application of established inverse-identification methods to a particular structural parameterization of cast-bronze cooling. It does not introduce a new least-squares algorithm or establish that the adopted structural parameterization is physically unique.

The objective of this study was to establish the regularities governing free linear shrinkage in cast multicomponent CuAl7Si2Sn1.5Mn0.3 aluminum bronze by reconstructing genetically successive structural states and identifying their effective integral-average coefficients of linear thermal contraction and structural-transformation components through a cascade solution of the inverse temperature–phase problem, as well as to assess the numerical and statistical stability of the resulting model.

## 3. Materials and Methods

The study was conducted on cast multicomponent aluminum bronzes of the Cu–Al–Si–Mn–Sn system, corresponding to the nominal alloy designation CuAl7Si2Sn1.5Mn0.3. Table 1 presents the adopted nominal composition range of the alloy [26] and the experimentally determined chemical compositions of the 17 investigated bronzes, together with their corresponding composition criterion *K_R_* values. The investigated compositions cover different combinations of the principal alloying elements within the specified composition range.

The composition criterion *K_R_*, defined by the previously proposed relationship [25,28], was used as a generalized descriptor of chemical composition:

(1) $$K_{R}=\left(1-\frac{nn}{100}\right)\frac{Al-Si-Mn}{{\left(1+Sn\right)}^{2}}$$

where Al, Si, Mn, Sn, and nn are the mass fractions of the alloying elements and impurities, wt.%.

In the investigated dataset, *K_R_* ranged from 0.41 to 0.85. Within this range, the set of thermal, structural, and deformation characteristics required for temperature–phase modeling was obtained.

Chemical composition was determined by optical emission spectroscopy (OES) using a SPECTROMAXx spectrometer (SPECTRO Analytical Instruments GmbH, Kleve, Germany). The temperatures of characteristic structural-phase transformations were determined by differential thermal analysis using an STA 449 C Jupiter instrument (NETZSCH-Gerätebau GmbH, Selb, Germany). The experimental procedure used for thermal analysis of the investigated bronze, including the heating and cooling conditions, was described previously [26].

Free linear shrinkage was determined using cylindrical specimens cast in quartz tubes with smooth inner surfaces according to the previously reported procedure [46]. After cooling, the specimens were held in air at 22 °C for 24–25 h before dimensional measurements. Their lengths were measured with an instrumental resolution of 0.001 mm. A total of 87 specimens representing 17 bronze compositions were tested, with 4–7 specimens per composition. Experimental free linear shrinkage was denoted by αᶠ,exp ≡ αB. For each bronze composition, the arithmetic mean free linear shrinkage was calculated from measurements of 4–7 specimens. The observed within-composition scatter was characterized by the half-range of the measured shrinkage values:

$$\Delta \alpha_{B,i}=\frac{\alpha_{B,i,max}-\alpha_{B,i,min}}{2},$$

where $\alpha_{B,i,max}$ and $\alpha_{B,i,min}$ are the maximum and minimum measured free-linear-shrinkage values among the specimens of composition i, respectively.

The half-ranges varied from 0.0028 to 0.0043 percentage points, with an average of 0.00347 percentage points. These values describe the observed spread of individual specimen measurements; they are not standard deviations, standard errors of the mean, or confidence intervals. They are distinct from the instrumental length-measurement resolution and the model-fitting residuals and are not used to establish the statistical significance of differences between composition means. Individual specimen measurements were unavailable for the present analysis; therefore, composition-specific standard deviations and confidence intervals could not be reconstructed.

Metallographic examinations were performed using a Neophot 21 optical microscope (Carl Zeiss Jena, Jena, Germany) at magnifications of ×125 and ×500. Quantitative metallographic analysis was performed on digital images of ten fields of view per specimen, and arithmetic mean fractions were calculated. The reported maximum relative field-to-field spread was 3.1% of the measured value for the total dark eutectoid region and 2.7% for the relative α-constituent fraction within that region. These summary spread values are not composition-specific standard deviations, standard errors, or confidence intervals and do not provide a complete uncertainty budget for the constituent fractions. In accordance with Delesse’s stereological principle, area fractions were used as estimates of the corresponding volume fractions of the structural constituents.

The quantitative analysis was performed at two magnification levels. At ×125, the fraction of the entire dark eutectoid structural region, *φ_D_*, was determined relative to the total field-of-view area. In the adopted structural interpretation, this region contained the eutectoid α constituent and the γ_2_ phase:

*φ_D_* = *φ_α,eut_* + *φ_γ_*_2_. (2)

Thus, *φ_D_* was not directly equated with the amount of the γ_2_ phase.

At ×500, the internal proportions of the constituents within the dark eutectoid region were determined. The relative fraction of the light eutectoid α constituent was calculated as:

*q_α,eut,i_* = *S_α,eut,i_*/*S_D,i_,* (3)

where *S_α,eut,i_* is the area of the light *α_eut_* constituent within the analyzed dark region, and *S_D,i_* is the total area of this region.

The relative fraction of the γ_2_ phase was determined from:

*q_γ_*_2_,*_i_* = 1 − *q_α,eut,i_.* (4)

Then, for composition *i*:

*φ_γ_*_2_,*_i_* = *φ_D_*_,*i*_*q_γ_*_2_,*_i_,* (5)

*φ_α,eut,i_* = *φ_D,i_q_α,eut,i_* = *φ_D,i_* − *φ_γ_*_2_,*_i_.* (6)

Thus, the total amount of the dark eutectoid region was determined at ×125, whereas its internal partitioning between the *α_eut_* and γ_2_ constituents was determined at ×500. This procedure quantitatively separated *φ_D_* into *φ_α,eut_* and *φ_γ_*_2_ without equating the entire optically dark region with the γ_2_ phase.

This partitioning was used only at the interpretation stage after the initial identification of the complex characteristics of the structural states. It was not an initial condition of the temperature–phase model and did not involve a priori assignment of intrinsic CLTE values to individual phases.

### 3.1. Adopted Physical and Mathematical Assumptions

The following physical and mathematical assumptions were adopted in developing the temperature–phase model of free linear shrinkage in the investigated bronzes.

1. The free linear shrinkage of the bronze was treated as the combined result of two components: thermal contraction of successive structural states and a structural-transformation strain contribution associated with changes in the structural-phase state during cooling.
2. The thermal component was determined over the full cooling range from the solidus temperature *T_S_* to room temperature *T*_0_. This temperature path was divided into three successive intervals bounded by the experimentally determined characteristic structural-phase transformation temperatures *T_S_*, *T_SU_*, *T_eut_*, and *T*_0_.
3. Each temperature interval was associated not with an individual crystallographic phase but with a structural state defined by the adopted genetic structure-formation pathway. Consequently, a formally identical phase composition produced by structural constituents of different origins was not regarded as sufficient evidence that their temperature–deformation characteristics were identical.
4. The temperature–deformation response of each structural state was described by an effective integral-average coefficient of linear thermal contraction (IA-CLTC). For a single-phase state, this coefficient characterized the integral-average response of the corresponding structural constituent; for a two- or multiphase state, it characterized the combined response of the structural constituents that coexist and interact mechanically within the corresponding temperature interval. The IA-CLTCs were identified from the combined experimental data on free linear shrinkage and the temperature intervals over which the structural states existed; they were not assigned a priori as tabulated properties of individual phases.
5. The temperature dependence of the instantaneous CLTE within an individual temperature interval was not specified explicitly. Its integral effect on the change in linear dimension was represented by the corresponding IA-CLTC of the structural state.
6. The equilibrium Cu–Al phase diagram was used as a pseudobinary approximation only for the qualitative physical interpretation of a possible sequence of phase transformations. The quantitative transition temperatures included in the mathematical model were determined directly from differential thermal analysis of the investigated bronzes.
7. The experimentally determined area fraction of the final dark eutectoid structural region, *φ_D_*, was not equated with the volume fraction of the γ_2_ phase. Its internal partitioning between the eutectoid *α_eut_* constituent and the γ_2_ phase was estimated by quantitative metallographic analysis at higher magnification using the adopted stereological approximation.
8. The deformation effect of a change in structural-phase state that was not reproduced by the thermal component of the model was represented by a separate structural-transformation term. This term was treated as an effective residual contribution of structural rearrangement to the final free linear shrinkage and was not attributed to a specific micromechanism of an individual phase transformation without independent experimental evidence.

Thus, the proposed model decomposes the final free linear shrinkage into thermal and structural-transformation components. The former describes the integral thermal contraction of genetically successive structural states over the corresponding temperature intervals, whereas the latter accounts for the residual deformation effect of structural-phase rearrangement not included in the thermal component.

### 3.2. Reconstruction of Genetic Structural States

The genetic structural states and the temperature limits of their existence were determined not by a single method but from a set of mutually consistent experimental and thermodynamic indicators. These included the thermal effects observed by differential thermal analysis, the nature and quantitative parameters of the microstructure, the compositional dependence of free linear shrinkage, and the phase relationships and sequence of equilibrium transformations in the Cu–Al phase diagram.

The combined use of these data provided the basis for distinguishing primary and secondary structural constituents. The subscript p (primary) was used for constituents attributed to the solidification stage; s (secondary), for constituents associated with a subsequent solid-state transformation; and eut, for constituents of the final eutectoid transformation.

Accordingly, the designations *α_p_*, *β_p_*, *α_s_*, and *β_s_* do not denote new crystallographic phases; they indicate the reconstructed origin of the corresponding structural constituents. The genetic pathways were subsequently used as interpretive structural-phase schemes consistent with the available observations and the qualitative Cu–Al reference, rather than as independently verified sequences of intermediate states.

On the basis of the combined experimental data, three genetically distinct temperature–phase pathways of structure formation were reconstructed over the investigated K_R_ range, as shown in Table 2.

Within the adopted reconstruction, the pathways differ in their assigned structural states and in the inferred origin of the constituents formed during solidification and subsequent cooling. Pathway I retains the primary *α_p_* constituent throughout the sequence of post-solidification states. In pathway II, a primary two-phase *α_p_* + *β_p_* state forms after solidification; subsequent cooling produces the secondary *α_s_* constituent. In pathway III, the primary *α_p_* constituent is absent: the initial post-solidification state is *β_p_*, from which the *α_s_* and eutectoid constituents form successively. The *α_s_* + *β_p_* subsystem in pathways II and III therefore shares a common *β_p_*-derived origin.

Within the adopted structural reconstruction, the bronzes with *K_R_* = 0.46 and *K_R_* = 0.45 were assigned to pathways II and III, respectively. This distinction represents an inferred change in the initial post-solidification state from *α_p_ + β_p_* to *β_p_*, rather than a directly observed phase boundary in the five-component alloy.

The exact position of this boundary within 0.45 < *K_R_* < 0.46 was not determined experimentally. Similarly, because no experimental free-linear-shrinkage values were available within 0.68 < *K_R_* < 0.73, the exact boundary between pathways I and II was not determined quantitatively.

The proposed genetic pathways are reconstructed temperature–phase schemes, rather than a directly established sequence of all intermediate crystallographic phases. The equilibrium Cu–Al diagram was used only as a qualitative reference within a pseudobinary interpretation; it does not quantitatively describe the phase equilibria of the Cu–Al–Si–Sn–Mn system. The characteristic thermal-event temperatures were measured directly for the investigated multicomponent bronzes, whereas their assignment to specific transformations remains interpretive. Optical metallography provides information on the final structural constituents but does not uniquely establish the identity and formation sequence of all intermediate high-temperature phases. In particular, the inferred change from *α_p_* + *β_p_* to *β_p_* between the compositions assigned to pathways II and III requires independent phase identification. The equal final shrinkage of these two bronzes does not validate this assignment.

Alternative intermediate transformation sequences compatible with the measured thermal events and final structural observations cannot be excluded. No comparative analysis demonstrating that the adopted pathways are unique or superior to such alternatives is presented.

An unchanged formal phase notation does not imply that the genetic structural states are identical. In particular, the reconstructed *α_p_* + *β_p_* → *α_p_* + *α_s_* + *β_p_* transition in pathway II formally remains an α + β state but, in the adopted genetic scheme, involves the formation of the secondary *α_s_* constituent.

The mathematical structure of the temperature intervals was the same for all bronzes, whereas their physical meaning was determined by the corresponding reconstructed genetic scheme.

Each two- or multiphase temperature interval was assigned a single effective IA-CLTC for the structural state as a whole, without a priori partitioning among the individual phases.

### 3.3. Thermal and Structural-Transformation Components

The experimentally determined free linear shrinkage was represented as the sum of two model components: thermal contraction of successive structural states and the residual deformation contribution of structural-phase rearrangement that is not reproduced by the thermal component.

For temperature interval *r* of bronze *i*, a scaled temperature coefficient was introduced:

*C_r,i_* = 10^−4^ · Δ*T_r,i_,* (7)

where the factor 10^−4^ converts an IA-CLTC expressed in 10^−6^ K^−1^ to shrinkage expressed as a percentage.

The thermal component was defined as:

(8) $$\alpha_{f,i}^{th}=\sum C_{r,i}\cdot {\bar{\alpha }}_{p,r}^{IA},\;\;\;\;r=1\ldots 3,$$

where ${\bar{\alpha }}_{p,r}^{IA}$ is the effective integral-average coefficient of linear thermal contraction of the structural state in interval r of genetic pathway p.

The residual deformation contribution associated with structural-phase rearrangement was represented in the following minimum-parameter form:

*α_tr,i_* = *k_p_·φ_i_,* (9)

where *k_p_* is the structural-transformation coefficient for the corresponding genetic pathway, percentage points per vol.%; and *φ_i_* is the structural parameter, vol.%.

The linear structural-transformation term was retained as a parsimonious baseline parameterization, rather than an experimentally established transformation law. A conditional comparison with a quadratic extension was performed for pathway III to examine whether a similar fit to integral shrinkage could coexist with a different component balance. This diagnostic comparison does not replace the baseline cascade identification.

The structural parameter was selected according to the reconstructed genesis of the final structural state: *φ_D_* was used for pathway I, whereas $\varphi_{\alpha ,eut}$ was used for pathways II and III. These parameters reflect the different physical meanings of the final structural-phase rearrangement within the adopted genetic schemes; *k_p_* was interpreted as an effective parameter of the corresponding pathway rather than as a universal characteristic of an individual phase.

A negative *k_p_* indicates that, within the model, the structural-transformation component acts in the direction opposite to thermal contraction and partly compensates for it. This result should not, by itself, be interpreted as direct evidence of macroscopic expansion during a specific phase transformation, but rather as a compensating contribution relative to the thermal component defined within the present model. It does not indicate a negative CLTE or IA-CLTC for any structural constituent.

The calculated free linear shrinkage was defined as:

(10) $$\alpha_{f,i}^{cal}=\sum_{r=1}^{3}C_{r,i}\cdot {\bar{\alpha }}_{p,r}^{IA}+k_{p}\varphi_{i},$$

or:

(11) $$\alpha_{f,i}^{cal}=\left[{\bar{\alpha }}_{p,1}^{IA}\cdot {\Delta T}_{1,i}+{\bar{\alpha }}_{p,2}^{IA}\cdot {\Delta T}_{2,i}+{\bar{\alpha }}_{p,3}^{IA}\cdot {\Delta T}_{3,i}\right]10^{-4}+k_{p}\varphi_{i,}$$

where i is the index of the investigated bronze; p is the structure-formation pathway; *r* is the temperature-interval index; *C_r,i_* is the temperature coefficient for interval *r* of pathway p; *k_p_* is the structural-transformation coefficient for pathway p; and *φ_i_* is the volume fraction of the structural constituent for bronze composition *i*.

Equation (10) has the same mathematical structure for all three pathways; the differences are determined by the physical meaning of the corresponding structural states, the IA-CLTC and *k_p_* values, and the choice of *φ_i_*.

Both contributions in Equation (10) are inferred jointly from the same set of final free-linear-shrinkage measurements; neither contribution is measured separately. Differential thermal analysis and quantitative metallography constrain the temperature intervals, reconstructed structural states, and structural predictors, but do not independently determine the strain assigned to either contribution. Accordingly, Equations (8)–(11) define an operational, model-dependent decomposition. The term *k_p_φ_i_* represents the effective residual contribution assigned to structural transformation under the adopted thermal parameterization and may also absorb discrepancies arising from that parameterization and the closure assumptions. Its identification therefore does not establish an independently measured transformation strain or a unique physical partition of the total shrinkage.

### 3.4. Cascade Inverse Identification

For each bronze, *T_S_*, *T_SU_*, *T_eut_*, $\alpha_{f}^{exp}$, and the corresponding structural parameter *φ_i_* were known experimentally. The characteristic temperatures were used to determine ΔT_1_, ΔT_2_, and ΔT_3_, after which Equation (10) provided one row of the parameter-identification system.

The inverse problem was written in matrix form:

y = A·x + ε,(12)

where y is the vector of experimental shrinkage values; A is the matrix of temperature and structural coefficients; x is the vector of identified parameters; and ε is the residual vector.

The baseline parameter estimates were obtained by least squares:

(13) $$\hat{x}=\arg\;\min{\left||A\cdot x-y|\right|}^{2}$$

The systems at the individual stages were overdetermined, and the numerical solution was obtained by QR or singular value decomposition (SVD) without explicitly computing (AᵀA)^−1^.

Identification was performed sequentially. For pathway I, the effective characteristic of the initial α_p_ state was fixed at 20.10 × 10^−6^ K^−1^, following the preliminary averaged estimate adopted in the original calculation. The coefficients of the remaining two structural states and k_I_ were estimated by least squares using the six pathway-I compositions. The six pathway-II compositions were subsequently used to identify the coefficients of its three structural states and k_II_.

After the initial identification, the complex characteristics of the final states of pathways I and II were decomposed for interpretation using the quantitatively determined proportions of the α and γ_2_ constituents. The resulting quantities were not interpreted as intrinsic phase CLTEs. In particular, the parameter assigned to the γ_2_ constituent was treated as a conditional effective IA-CLTC of the γ_2_ constituent within the adopted decomposition, rather than as a fundamental temperature-dependent characteristic of the γ_2_ phase.

To close the pathway-III problem, the genetic relationship between its intermediate *α_s_* + *β_p_* state and the *β_p_*-derived subsystem of the intermediate *α_p_* + *α_s_* + *β_p_* state in pathway II was used. The effective IA-CLTC of the *α_s_* + *β_p_* subsystem was extracted from the complex characteristic of the latter state by accounting for the previously determined characteristic of the primary *α_p_* constituent and the quantitative proportions of the constituents.

For closure of the pathway-III problem, the effective temperature–deformation characteristic of the genetically related *α_s_* + *β_p_* subsystem was assumed to remain unchanged when transferred from pathway II. This modeling assumption is required to reduce the dimensionality of the final stage of the inverse problem and requires independent experimental verification.

Under this assumption, the IA-CLTC of the intermediate state in pathway III was no longer treated as an independent unknown parameter. The IA-CLTC of the initial *β_p_* state and the coefficient k_III_ were jointly determined from five pathway-III bronzes.

The overall cascade-identification sequence was:

I → II → decomposition of the final states → effective characteristic of (*α_s_* + *β_p_*) → III.

Thus, information was transferred between stages on the basis of the adopted genetic relationship between structural constituents rather than formal identity of phase composition. This reduced the number of simultaneously identified parameters but also constitutes one of the modeling assumptions of the proposed approach.

### 3.5. Assessment of the Numerical and Statistical Stability of the Model

High accuracy in reproducing integral free linear shrinkage was not regarded as sufficient evidence that the individual internal parameters were unique. Therefore, the numerical, algorithmic, and statistical stability of the inverse problem was assessed in addition to obtaining the baseline least-squares solution.

Three distinct aspects were considered. Within a fixed linear identification stage, full column rank establishes uniqueness of the parameter vector for the specified inputs and closure assumptions. This stage-specific algebraic identifiability does not establish uniqueness of the reconstructed structural schemes or physical mechanisms. Practical identifiability concerns the sensitivity of parameter estimates to finite sampling, input perturbations, and dependence between the effects of different parameters. Predictive performance concerns shrinkage estimates for compositions excluded from parameter estimation and must be distinguished from reproduction of the identification dataset.

Sensitivity to the choice of optimization criterion was examined using least absolute deviations (LAD), minimax optimization, and nonnegative least squares (NNLS). LAD minimized the sum of absolute residuals, whereas minimax optimization minimized the maximum absolute residual.

In NNLS, nonnegativity constraints were imposed on the IA-CLTC temperature–deformation parameters. The signs of the structural-transformation coefficients *k_p_* were not subject to a nonnegativity constraint because a negative value has a distinct meaning in the model.

Additional conditional sensitivity analysis was performed for the auxiliary decomposition of the final states of pathways I and II. Experimental constituent fractions were used in the 12-equation least-squares system, with the complex final-state IA-CLTCs fixed at 18.99 and 11.57 × 10^−6^ K^−1^, respectively. The unknowns were the effective characteristics of the combined α constituent in each pathway and a shared effective γ_2_ characteristic. Each composition was omitted in turn, and the three coefficients were re-estimated by unconstrained least squares. Minimum and maximum estimates across the twelve omissions were reported as conditional sensitivity ranges. These ranges are not confidence intervals and do not include uncertainty in the fixed complex final-state characteristics.

The numerical identifiability of the systems was assessed by SVD using the matrix rank, singular values, and condition number.

For pathway III, conditioning was evaluated both for the original two-column identification matrix and after dividing each column by its Euclidean norm without centering. The cosine similarity of the normalized columns was also calculated. The unscaled condition number depends on the units and magnitudes of the regressors; the normalized value was therefore used to assess their near-collinearity. A separate conditional leave-one-out analysis of the pathway-III stage was performed by omitting each of its five compositions in turn and re-estimating the two unknown parameters while holding the characteristics supplied by the preceding cascade stages fixed.

Conditional correlation between the two pathway-III parameter estimates was evaluated from the ordinary least-squares covariance structure for the fixed identification matrix, assuming independent residual errors with a common variance. The covariance matrix is proportional to the inverse of the regressor Gram matrix; the proportionality factor cancels when calculating correlation. This analytical correlation is distinct from both the cosine similarity of the matrix columns and correlations that would be obtained from full-cascade Monte Carlo or bootstrap realizations.

Parameter sensitivity to individual experiments was evaluated by leave-one-out analysis. According to the original computational summary, cascade Monte Carlo analysis used independent additive normally distributed perturbations of the measured shrinkage, with zero mean and standard deviations of 0.00780, 0.00164, and 0.00553 percentage points for pathways I, II, and III, respectively. These scales were based on pathway-specific fitting RMSEs rather than independently measured repeatability uncertainties. In each of 200,000 realizations, the dependent cascade stages were repeated, including the final-state decomposition and re-estimation of the transferred subsystem characteristic; the initial αp-state reference remained fixed at 20.10 × 10^−6^ K^−1^. Transformation temperatures and metallographic fractions were not jointly perturbed. The resulting percentile intervals therefore describe sensitivity to this assumed shrinkage-perturbation model, rather than a complete propagation of experimental measurement uncertainty.

Sensitivity to the limited pathway-III dataset was assessed by a new conditional pairs-bootstrap analysis. All 5^5^ = 3125 equally probable ordered samples of size five with replacement were enumerated from the five pathway-III compositions. Complete composition records were resampled together, preserving the pairing of measured shrinkage, temperature intervals, and structural predictors. Repeated records were permitted, and different orderings were retained with their corresponding multiplicities. Exhaustive enumeration required neither a random-number generator nor a random seed.

The upstream characteristics were held fixed. The transferred intermediate-state IA-CLTC was 36.654 × 10^−6^ K^−1^, and room temperature was 22 °C. The final-state coefficients and structural predictors were reconstructed from the rounded published inputs using the same expressions as in the conditional functional-form analysis described below. For each resample, the initial *β_p_*-state IA-CLTC and k_III_ were re-estimated by unconstrained least squares. The two columns of the identification matrix were 10^−4^(T_S,i_ − T_SU,i_) and φ_i_; the response was the measured shrinkage after subtraction of the fixed intermediate- and final-state thermal contributions.

For rank testing, each matrix column was divided by its Euclidean norm. A resample was retained only if its smallest singular value exceeded τ = 5εσmax, where ε = 2.220446049250313 × 10^−16^ is double-precision machine epsilon and σmax is the largest singular value of the normalized matrix. Exactly five resamples, each containing five copies of the same composition, were excluded as rank deficient. Thus, 3120 resamples were retained and five were excluded, corresponding to an exclusion rate of 0.16%. No additional exclusions based on parameter signs, condition numbers, or residual errors were applied. Least-squares solutions were obtained using numpy.linalg.lstsq with rcond = None, and the 2.5th and 97.5th percentiles were calculated using numpy.quantile with method = ‘inverted_cdf’ in NumPy 2.3.5. The calculation script and complete acceptance log are provided in Supplementary File S1. These intervals describe conditional sensitivity to resampling the five pathway-III compositions; they do not propagate uncertainty in preceding cascade stages or experimental measurement uncertainty.

A separate conditional sensitivity analysis was performed to assess the influence of the coefficient transferred from pathway II to pathway III. The effective IA-CLTC of the *α_s_ + β_p_* subsystem was varied by −20%, −10%, −5%, +5%, +10%, and +20% relative to the baseline value of 36.654 × 10^−6^ K^−1^. These variations were selected as sensitivity scenarios rather than experimentally established uncertainty bounds. For each scenario, the effective IA-CLTC of the initial *β_p_* state and the structural-transformation coefficient k_III_ were re-estimated by least squares using the five pathway-III compositions. All other inputs, including the temperature intervals, structural predictors, and final-state IA-CLTCs, were held fixed.

Changes relative to the baseline least-squares solution were calculated using the rounded temperature. The structural predictor φ_α_,eut was calculated from Equations (6) and (15). Because the pathway-III model is linear in the two re-estimated parameters and the transferred coefficient, their changes can be determined directly from the perturbed least-squares system while the fixed final-state contribution cancels. The analysis reports changes in the identified parameters and the maximum absolute change in calculated shrinkage across the five compositions. For comparison, shrinkage changes were also calculated with both identified parameters held fixed. This analysis isolates the transfer assumption and does not represent uncertainty propagation through the entire cascade.

For the component-level assessment, the thermal and structural-transformation contributions at *K_R_* = 0.45 were recalculated in each transfer-coefficient scenario using the rounded-input reconstruction of the final-state IA-CLTC specified below for the functional-form comparison. The corresponding pathway-II contributions at *K_R_* = 0.46 were held unchanged.

Functional-form sensitivity was examined conditionally for the five pathway-III compositions by comparing αtr,i = k_III_φi with αtr,i = k_III_φi + c_III_φi^2^. The transferred intermediate-state IA-CLTC was fixed at 36.654 × 10^−6^ K^−1^ and room temperature at 22 °C. Structural predictors were calculated from Equations (6), (14) and (15). For this diagnostic calculation, the final-state IA-CLTC was reconstructed from the rounded effective constituent characteristics as a3,i = 11.24(1 – fγ_2_,i) + 12.50fγ_2_,i, where fγ_2_,i = φD,iqγ_2_,i/100 and qγ_2_,i is expressed as a fraction.

The initial *β_p_* IA-CLTC and the structural-transformation coefficients were re-estimated while all upstream characteristics remained fixed. The quadratic fit imposed a nonnegative *β_p_* IA-CLTC, a nonpositive structural-transformation contribution, and a nonpositive derivative of that contribution throughout the observed structural-fraction range. These constraints express the adopted compensating interpretation, not universal physical laws. The constrained least-squares solution was obtained by enumerating active constraint sets. The comparison evaluates fitting and decomposition sensitivity within pathway III; it is neither full-cascade cross-validation nor independent physical validation.

After parameter identification, back-calculation was performed for all 17 bronzes using Equation (10). The calculated and experimental values were compared using R^2^, RMSE, MAPE, and the maximum absolute relative error. The quality of reproduction of integral shrinkage and the statistical identifiability of individual internal parameters were treated as fundamentally different characteristics of the inverse problem.

In each fold, the intercept and slope were estimated from 16 compositions, and shrinkage was predicted for the omitted composition using its *K_R_* value. The pooled held-out predictions were evaluated using RMSE, MAPE, and *Q*^2^, defined as $Q^{2}=1-\frac{\sum_{i=1}^{17}{\left(y_{i}-\hat{y_{i,-i}}\right)}^{2}}{\sum_{i=1}^{17}{\left(y_{i}-\bar{y}\right)}^{2}}$, where $\hat{y_{i,-i}}$ denotes the prediction obtained without composition i.

This benchmark cross-validation is distinct from the conditional cascade LOOCV reported in Section 4.4. The latter re-estimates dependent cascade stages within each fold while holding the adopted reference IA-CLTC fixed; it therefore does not propagate uncertainty in the original determination of that reference coefficient and is not external validation on new alloys.

## 4. Results

Comparison of the differential thermal analysis, quantitative metallography, and experimental free-linear-shrinkage data showed that changes in the composition criterion Kᵣ were accompanied by simultaneous changes in the temperatures of characteristic structural-phase transformations, the quantitative proportions of structural constituents, and the final strain. In accordance with the reconstructed genetic structure-formation schemes, the investigated bronzes were divided into three groups. The numbers of specimens tested for each bronze composition and the corresponding reported errors of the mean free linear shrinkage are presented in Table 3. The experimentally determined thermal, structural, and deformation characteristics of the investigated bronzes are summarized in Table 4.

As a parsimonious benchmark, the linear relationship in Equation (16) was evaluated using both full-data fitting and leave-one-composition-out cross-validation on the 17 compositions in Table 4.

For the 17 investigated bronzes in the range *K_R_* = 0.41–0.85, the area fraction of the final dark eutectoid structural constituent *φ_D_* increased from 11.5% to 58.1% as the composition criterion decreased, whereas the experimentally determined free linear shrinkage decreased from 1.77% to 1.37%.

Quantitative analysis of the internal structure of the dark eutectoid region revealed a systematic change in the proportions of its constituents with *K_R_*. The experimental data for the relative fraction of the γ_2_ constituent were described by:

(14) $$q_{\gamma 2,i}\;0.71\;-\;0.777K_{R,i}\;+\;1.23K_{R,i}^{2}.$$

from which the relative fraction of the light eutectoid α constituent is:

(15) $$q_{\alpha ,eut,i}=1-q_{\gamma_{2,i}}=0.29+0.777K_{R,i}-1.23K_{R,i}^{2}.$$

Table 5 presents the mean experimental metallographic fractions and their derived constituent fractions for all 17 compositions. The relative γ_2_-constituent fraction refers to the interior of the dark eutectoid region, whereas φ_α,eut,i_ and φ_γ2,i_ refer to the entire microstructure. The latter quantities were calculated from the experimental fractions, not from Equation (14). The constituent labels retain the interpretive status described in Section 3.2.

Evaluating Equation (14) against the 17 experimental relative γ_2_-constituent fractions, without rounding the calculated predictions before computing residuals, gave *R^2^* = 0.99403, RMSE = 0.919 percentage points, and MAE = 0.711 percentage points. The maximum absolute residual was 2.415 percentage points at *K_R_* = 0.46. Residuals were evaluated on the percentage scale of q_γ2_, rather than as fractions of the entire microstructure. These statistics describe agreement with the fitted dataset and are not estimates of metallographic measurement uncertainty or independent validation of the constituent assignments.

The overall macroscopic dependence of experimental free linear shrinkage on the composition criterion over the investigated range was approximated by the linear relationship:

(16) $$\alpha_{f}^{reg}=0.99587+0.91079K_{R,i}$$

Thus, free linear shrinkage increases linearly with *K_R_* over the entire investigated compositional range of the bronze.

Further temperature–phase analysis nevertheless shows that this smooth macroscopic relationship is realized through the successive occurrence of three genetically distinct structure-formation pathways. At the same time, *T_S_*, *T_SU_*, and *T_eut_* vary differently with composition. Thus, a change in *K_R_* is accompanied not only by a quantitative change in the final structure but also by a change in the temperature spans of the individual stages of its formation.

### 4.1. Identification of Effective IA-CLTCs of Structural States

The sequential solution of the cascade inverse problem identified the effective IA-CLTCs of the structural states in the three genetic pathways.

For pathway I, the sequence obtained was: 20.10 → 15.44 → 18.99 × 10^−6^ K^−1^.

For pathway II: 21.88 → 29.40 → 11.57 × 10^−6^ K^−1^.

For pathway III, the identified IA-CLTC of the initial *β_p_* state was 25.90 × 10^−6^ K^−1^; the intermediate *α_s_ + β_p_* state was represented by the transferred effective value of 36.60 × 10^−6^ K^−1^, whereas the composition-dependent IA-CLTC of the final state ranged from 11.662 to 11.679 × 10^−6^ K^−1^.

The consolidated parameter-identification results are given in Table 6.

The largest differences between pathways were found for the intermediate temperature interval ΔT_2_. For pathway I, the IA-CLTC of the *α_p_* + *β_s_* state was 15.44 × 10^−6^ K^−1^, whereas the value for the *α_p_* + *α_s_* + *β_p_* state in pathway II was 29.40 × 10^−6^ K^−1^. A value of 36.60 × 10^−6^ K^−1^ was obtained for the genetically related *β_p_*-derived *α_s_* + *β_p_* subsystem used to close the pathway-III problem. Thus, the most pronounced change in the temperature–deformation response occurs at the intermediate stage of structure formation.

The complex IA-CLTCs of the final structural states in pathways I and II were further decomposed into effective characteristics of the combined α and γ_2_ constituents using their quantitatively determined proportions. The resulting effective IA-CLTCs were 19.97 × 10^−6^ K^−1^ for the combined α constituent in the final state of pathway I, 11.24 × 10^−6^ K^−1^ for the combined genetically heterogeneous α constituent in pathway II, and 12.50 × 10^−6^ K^−1^ for the γ_2_ constituent. These quantities are parameters of the adopted interpretive decomposition and are not regarded as intrinsic CLTEs of the corresponding phases.

The influence of using Equation (14) was examined by repeating the auxiliary least-squares decomposition for the 12 pathway-I and pathway-II compositions using the experimental constituent fractions. The identified complex final-state IA-CLTCs were held fixed at 18.99 and 11.57 × 10^−6^ K^−1^, respectively. Replacing the fitted fractions with the experimental estimates changed the effective combined α characteristics by +0.10% for pathway I and +0.43% for pathway II, and the effective γ_2_ characteristic by −1.12%. Thus, smoothing the fractions using Equation (14) has a limited effect on these three auxiliary estimates under the fixed-state assumptions. This comparison neither propagates metallographic uncertainty through the entire cascade nor establishes the reliability of all pathway-III parameters.

The effective combined α characteristic in pathway I, 19.97 × 10^−6^ K^−1^, differs from the adopted initial-state reference of 20.10 × 10^−6^ K^−1^ by approximately 0.65%. This numerical agreement is consistent with the adopted representation but is not an independent validation of either characteristic.

### 4.2. Structural-Transformation Contribution and Reproduction of Free Linear Shrinkage

In addition to the IA-CLTCs, structural-transformation coefficients were identified for the three genetic pathways:

k_I_ = −0.00645; k_II_ = −0.00732; k_III_ = −0.01615 percentage points per vol.%.

The absolute *k_p_* values are similar for pathways I and II, whereas the magnitude of the coefficient increases by a factor of approximately 2.21 from pathway II to pathway III. The negative sign of *k_p_* indicates that, within the adopted model, the structural-transformation component acts in the direction opposite to thermal contraction and partly compensates for it.

Back-calculation for all 17 investigated bronzes showed close agreement between the calculated and experimental free-linear-shrinkage values throughout the range *K_R_* = 0.41–0.85. For the complete dataset:

R^2^ = 0.998; RMSE = 0.00562 percentage points; MAPE = 0.266%; *δ_max_* = 0.75%.

**Figure 1:**
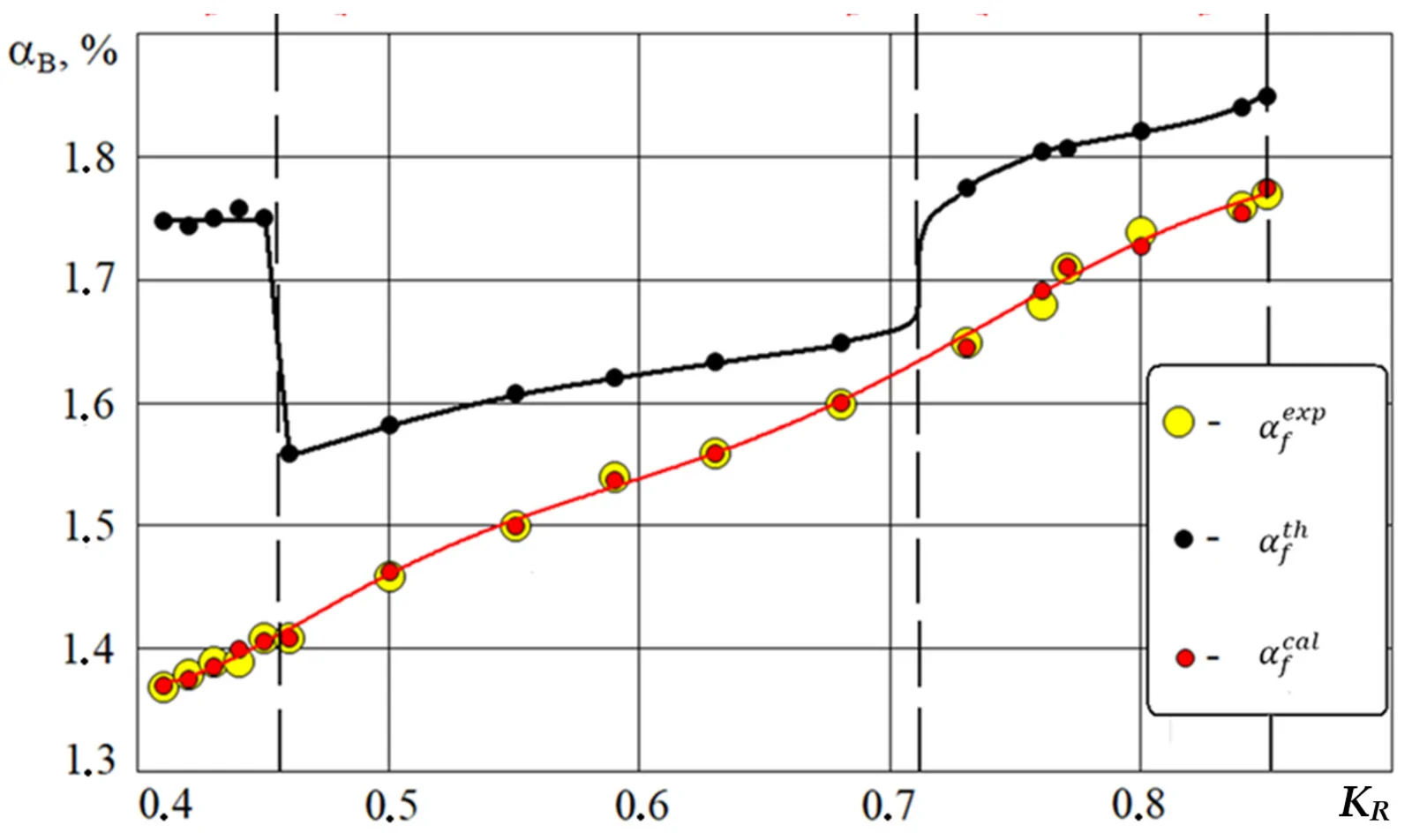
Dependence of the experimental $\alpha_{f}^{exp}$, thermal $\alpha_{f}^{th}$, and calculated $\alpha_{f}^{cal}$ components of free linear shrinkage on *K_R_* for the three genetic phase pathways. The thermal contribution is a model-dependent estimate; the calculated total shrinkage is the sum of the thermal and structural-transformation terms. Neither term was measured independently.

Thus, the temperature–phase model reproduces the experimental integral free linear shrinkage with high accuracy within the dataset used for parameter identification. This result characterizes the model’s internal reproduction capability and does not constitute independent external validation, as illustrated by the relationships in Figure 1.

Comparison with the linear benchmark is presented in Table 7. Refitting Equation (16) to the rounded Table 4 data gave *R*^2^ = 0.99671, RMSE = 0.00818 percentage points, and MAPE = 0.419%. Leave-one-composition-out cross-validation gave *Q*^2^ = 0.99589, RMSE = 0.00915 percentage points, and MAPE = 0.467%. Thus, the simple regression already provides accurate held-out estimates within the sampled compositional range. The lower full-data fitting error of the inverse model does not establish an improvement in prediction of omitted compositions.

As shown in Figure 1, the $\alpha_{f}^{exp}$(*K_R_*) and $\alpha_{f}^{cal}$(*K_R_*) relationships nearly coincide and remain smooth over the entire investigated *K_R_* range. The thermal component $\alpha_{f}^{th}$ behaves fundamentally differently, reflecting the transition between genetic phase pathways. The most pronounced change occurs between the bronzes with *K_R_* = 0.46 and *K_R_* = 0.45: the thermal component increases from 1.5593% to 1.7518%, that is, by +0.1925 percentage points, whereas the structural-transformation component changes from −0.1505 to −0.3456 percentage points, that is, by −0.1951 percentage points. Their resultant change is only −0.0026 percentage points, corresponding to a change in calculated free linear shrinkage from 1.4088% to 1.4062%, whereas the experimental value is 1.41% for both bronzes.

The calculated difference of −0.0026 percentage points between the two compositions is smaller in magnitude than the experimental shrinkage half-ranges of 0.0032 and 0.0030 percentage points reported for *K_R_* = 0.46 and *K_R_* = 0.45, respectively (Table 3). However, these half-ranges describe the spread of individual specimen measurements and do not quantify the uncertainty of the composition means. Their comparison with the calculated difference therefore does not establish statistical significance or its absence. The difference is also smaller than the 0.01 percentage-point reporting increment of the experimental means. Accordingly, it is interpreted as a small difference between model predictions rather than an experimentally established difference in free linear shrinkage.

Thus, within the adopted model, the nearly continuous macroscopic *α_f_* = f(*K_R_*) relationship is compatible with different calculated partitions of shrinkage between the thermal and structural-transformation terms. At the boundary between pathways II and III, these terms exhibit nearly compensating changes. This is a result of the fitted decomposition conditional on the reconstructed pathways and the pathway-III closure assumption; it should not be interpreted as independent experimental evidence of an abrupt change in the physical shrinkage mechanism.

### 4.3. Redistribution of Deformation Contributions Among Temperature Intervals

**Figure 2:**
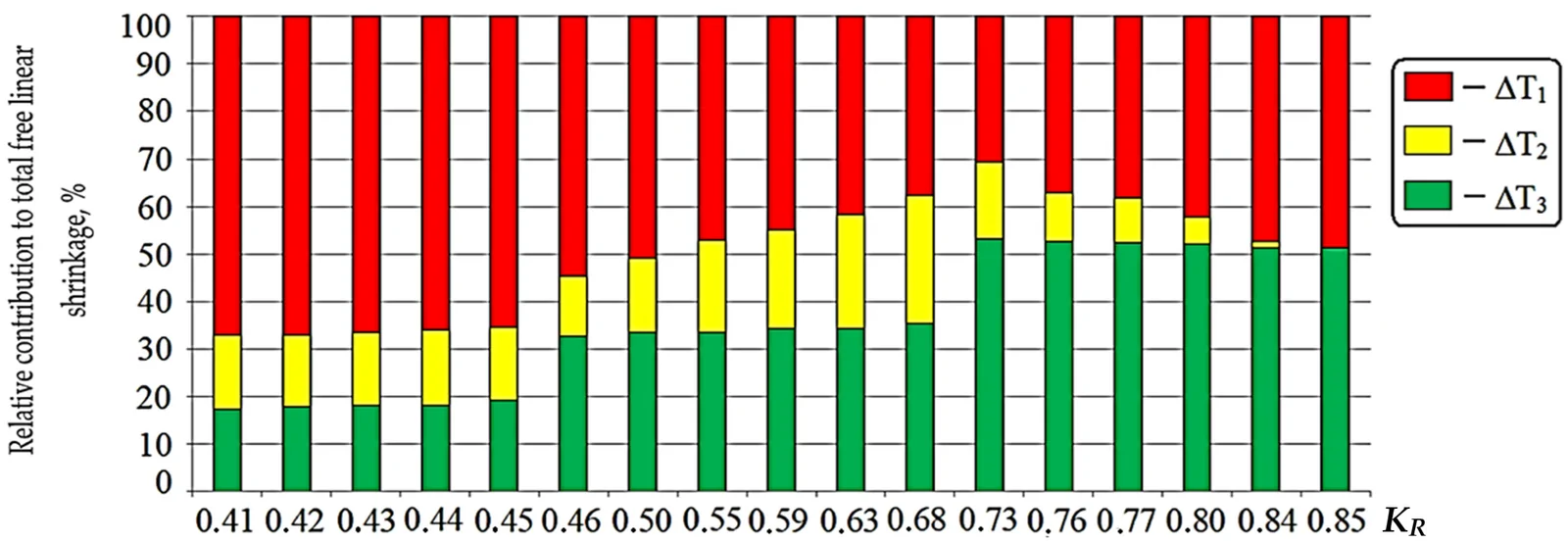
Distribution of the relative contributions of the temperature intervals to the calculated free-linear-shrinkage balance as a function of the composition criterion $K_{R}$; the integral structural-transformation contribution is formally included in the resultant ΔT_3_ contribution for comparative representation.

Separate calculation of the contributions from the three temperature intervals showed a substantial change in their relative proportions at transitions between genetic pathways. Because the structural-transformation component $k_{p}\varphi_{i}$ was identified from the final free linear shrinkage as an integral residual contribution, the available experimental data do not permit its direct allocation among ΔT_1_, ΔT_2_, and ΔT_3_. Therefore, the structural-transformation contribution cannot be physically localized within a specific temperature interval. For the comparative representation in Figure 2, this integral residual contribution was formally combined with the lower-temperature contribution ΔT_3_ solely to close the calculated deformation balance. This representation should not be interpreted as evidence that the structural-transformation strain physically develops within ΔT_3_. Figure 2 therefore illustrates the redistribution of the calculated deformation balance under this adopted representation.

For pathway-I bronzes, the lower temperature interval ΔT_3_ makes the largest contribution to the resultant strain, accounting for approximately 51–53%. For pathway II, the resultant contribution of this interval decreases to 33–35%, while a gradual redistribution between ΔT_1_ and ΔT_2_ occurs within the pathway. The pattern changes fundamentally for pathway III: 65.5–67.1% of the calculated final strain is associated with the upper temperature interval ΔT_1_, approximately 15–16% with ΔT_2_, and only 17–19% with the resultant contribution of ΔT_3_.

The most abrupt redistribution again occurs at the boundary between pathways II and III. Across the *K_R_* = 0.46 → 0.45 transition, the contribution of ΔT_1_ increases from approximately 54.7% to 65.6%, whereas the resultant contribution of ΔT_3_ decreases from 32.8% to 19.2%. The experimental final shrinkage of both bronzes nevertheless remains the same at 1.41%.

Thus, a change in *K_R_* is accompanied not only by a change in final free linear shrinkage but also by a substantial redistribution of its calculated deformation balance among the temperature intervals. The secondary structural constituent is not an independent cause of the shrinkage change; it is both a product and an indicator of a change in the genetic temperature–phase pathway, which determines the sequence of structural states, their IA-CLTCs, the temperature intervals over which they exist, and the structural-transformation contribution.

### 4.4. Numerical and Statistical Stability of Parameter Identification

Evaluation using the alternative LS, LAD, minimax, and NNLS criteria showed that changing the objective function did not fundamentally alter the central estimates of the principal effective parameters. The maximum differences in the estimates of the combined α constituent in pathway I, the α constituent in pathway II, and the conditional γ_2_ contribution were approximately 1.2%, 3.3%, and 3.2%, respectively, relative to the baseline LS estimates.

SVD analysis confirmed full rank of the identification systems. The normalized condition number reported for the pathway I–II system was 10.55. The pathway-III matrix had rank 2, with an unscaled condition number of approximately 2.14 × 10^4^. After normalization of each column to unit Euclidean norm without centering, its condition number was 67.88. The cosine similarity between the normalized columns was approximately 0.9996, indicating near-collinearity in the adopted system without an intercept. Thus, full rank permits a unique least-squares solution for the fixed system but does not ensure low sensitivity of the individual parameter estimates.

Under the fixed-matrix, independent equal-variance error assumption, the conditional correlation between the estimated *β_p_* IA-CLTC and k_III_ is −0.999566. The near-perfect negative correlation indicates that their effects on the fitted shrinkage can largely compensate for one another. Consequently, a well-reproduced total response does not imply precise separate determination of these parameters. This value is an analytical conditional diagnostic, not an empirical correlation calculated from the reported Monte Carlo or bootstrap distributions.

Conditional leave-one-out analysis identified the composition with *K_R_* = 0.45 as the most influential of the five pathway-III observations. Its omission reduced the estimated IA-CLTC of the initial *β_p_* state by approximately 16.1% and the magnitude of k_III_ by approximately 40.2%. These changes characterize sensitivity to individual observations at the final identification stage, with the upstream characteristics held fixed.

The conditional sensitivity results for the transferred pathway-III coefficient are presented in Table 8. A ±20% variation changed the identified IA-CLTC of the initial *β_p_* state by approximately ∓1.449 × 10^−6^ K^−1^, corresponding to ∓5.59% of its reported baseline value. The magnitude of k_III_ changed by approximately ∓2.36%. After re-estimation of both parameters, the maximum absolute change in calculated shrinkage was approximately 0.00131 percentage points. For ±10% and ±5% variations, the corresponding maximum changes were approximately 0.000657 and 0.000329 percentage points, respectively.

The maximum absolute shrinkage changes reported in Table 8 represent deviations from the baseline calculated shrinkage after re-estimation of the pathway-III parameters and should not be confused with the fitting RMSE values, which quantify deviations between calculated and experimental shrinkage. For variations of −20, −10, −5, 0, +5, +10, and +20% in the transferred IA-CLTC, the corresponding fitting RMSE values were 0.00477, 0.00515, 0.00534, 0.00553, 0.00573, 0.00592, and 0.00632 percentage points, respectively.

When the identified *β_p_* IA-CLTC and k_III_ were instead held fixed, the maximum absolute changes in calculated shrinkage were 0.04450, 0.02222, and 0.01111 percentage points for ±20%, ±10%, and ±5% variations, respectively. The substantially smaller changes after re-estimation show that the two identified parameters partly compensate for variation in the transferred coefficient. These results quantify the conditional sensitivity of the pathway-III solution; they do not independently validate the transferred value.

The component-level calculation using rounded tabulated inputs showed that, across the ±20% transfer-coefficient scenarios with both pathway-III parameters re-estimated, the thermal contribution at *K_R_* = 0.45 ranged from 1.7432% to 1.7604%, the structural-transformation contribution ranged from −0.3538 to −0.3374 percentage points, and their sum ranged from 1.4058% to 1.4067%. Relative to the unchanged pathway-II result at *K_R_* = 0.46, the thermal increase ranged from 0.1839 to 0.2011 percentage points, accompanied by a structural-transformation change ranging from −0.2033 to −0.1869 percentage points. Thus, nearly compensating component changes persist in the investigated transfer-coefficient scenarios within the adopted linear parameterization. This conditional result does not establish an experimentally verified compensation mechanism or robustness to alternative functional forms.

Table 9 summarizes the previously reported cascade Monte Carlo intervals and the newly computed conditional pathway-III bootstrap intervals. The Monte Carlo results were obtained from 200,000 realizations. The new bootstrap analysis enumerated 3125 ordered resamples, of which 3120 were retained and five were excluded by the rank criterion specified in Section 3.5. The bootstrap results are conditional on fixed upstream characteristics and should not be interpreted as full-cascade uncertainty estimates.

The new conditional bootstrap interval for k_III_ crosses zero, showing that the negative baseline estimate is not stable under all retained resamples of the five pathway-III compositions. The corresponding interval for the initial *β_p_*-state IA-CLTC remains positive in this analysis. This does not establish full-cascade stability because the upstream characteristics were held fixed.

Together with the conditioning and leave-one-out results, these findings indicate sensitivity of the separate pathway-III parameter estimates to the limited sample. The transfer-coefficient analysis and the bootstrap analysis examine different perturbations: the former varies a transferred characteristic while retaining the experimental sample, whereas the latter resamples the composition records while retaining the upstream characteristics. Both analyses remain conditional on the adopted structural schemes and identification procedures and do not independently validate the pathways or the physical decomposition of shrinkage.

The conditional functional-form comparison is summarized in Table 10. Using rounded published inputs, the linear and constrained quadratic forms gave similar fitting RMSEs of 0.005532 and 0.005442 percentage points, respectively. Nevertheless, the identified *β_p_* IA-CLTC changed from 25.9042 to 16.1963 × 10^−6^ K^−1^, and the calculated component balance changed substantially. Thus, a similar integral fit does not uniquely determine the separate contributions.

The constrained quadratic solution satisfies the imposed sign and monotonicity restrictions but reaches a constraint boundary. Its slightly smaller fitting error does not establish greater physical validity or predictive performance. The comparison supports treating the separate contributions as parameterization-dependent estimates and retaining the linear form as a simpler baseline rather than a uniquely validated physical law.

To evaluate the predictive stability of the adopted structural-transformation parameterization, conditional cascade leave-one-composition-out cross-validation (LOOCV) was performed for the original linear model and two quadratic extensions: an unconstrained quadratic model and a fully constrained quadratic model. In each validation cycle, one of the 17 investigated bronze compositions was excluded, and the dependent stages of cascade parameter identification were repeated using the remaining data. The effective IA-CLTC of the primary α_p_ state in genetic pathway I was held fixed at 20.10 × 10^−6^ K^−1^ throughout the procedure. Consequently, the resulting cross-validation metrics characterize predictive performance conditional on the adopted reference coefficient and do not include uncertainty associated with its initial estimation. The results are summarized in Table 11.

As shown in Table 11, the original linear parameterization provides the lowest cross-validation RMSE for each of the three genetic pathways and for the complete dataset. The overall RMSE of the linear model is 0.009953 percentage points, compared with 0.032328 percentage points for the unconstrained quadratic model and 0.017027 percentage points for the fully constrained quadratic model. Thus, introducing an additional quadratic term increases the overall cross-validation error by approximately 3.25 and 1.71 times, respectively.

The largest deterioration in predictive performance is observed for genetic pathway III, where the RMSE increases from 0.009213 percentage points for the linear model to 0.045690 percentage points for the unconstrained quadratic model. Imposing the adopted constraints reduces this error to 0.015016 percentage points, although it remains higher than that of the linear parameterization. These results indicate that increasing the complexity of the structural-transformation term does not improve the predictive stability of the cascade model for the investigated dataset.

Therefore, the original linear parameterization is retained as the preferred baseline model because it provides the lowest conditional cross-validation error among the examined alternatives while requiring fewer adjustable parameters. However, this result does not establish that the structural-transformation contribution is physically linear or that the reconstructed thermal and structural-transformation components are uniquely determined. Their quantitative separation remains dependent on the adopted temperature–phase reconstruction and model parameterization.

## 5. Discussion

The experimental results show that final free linear shrinkage varies with composition together with the measured transformation temperatures and final structural parameters. Within the reconstructed genetic pathways, the inverse model represents these relationships through changes in the effective coefficients and the calculated thermal and structural-transformation contributions. This interpretation is conditional on the adopted structural schemes and parameterization and does not constitute a direct experimental reconstruction of the separate strain contributions.

An IA-CLTC is an effective coefficient assigned to a reconstructed structural state over a specified temperature interval. Structural evolution is represented by the succession of these states and their interval boundaries, rather than by explicitly resolving continuously changing phase fractions and local strains. For a multiphase state, the fitted coefficient may indirectly reflect the combined influence of constituent properties, phase proportions, morphology, spatial connectivity, and internal mechanical constraints. However, the present model does not explicitly solve constituent-level stress equilibrium, strain compatibility, or relaxation and cannot separate the contributions of these mechanisms.

Changes in temperature interval, alloy composition, cooling history, or phase morphology may therefore change the effective response represented by an IA-CLTC. These dependencies were not independently quantified in this study. The coefficients should consequently be interpreted as conditional parameters of the adopted reconstruction and parameterization, rather than transferable material constants. In particular, their interval-averaged form does not establish equivalence to an independently measured average CLTE. Application to different cooling conditions or structural morphologies requires re-identification or independent validation.

The close agreement between the adopted reference IA-CLTC of the primary αp state in pathway I (20.10 × 10^−6^ K^−1^) and the subsequently derived effective coefficient of the combined α constituent (19.97 × 10^−6^ K^−1^) supports internal consistency of the adopted parameterization, but is not an independent validation of the reference value. Both values are of the same order of magnitude as published thermal-expansion characteristics of Cu–Al alloys [37,38].

This result also defines the limits of a phase-based interpretation of the identified parameters. The value of 12.50 × 10^−6^ K^−1^ assigned to the γ_2_ constituent is its conditional effective characteristic within the adopted decomposition, whereas the combined parameter of the α constituent in pathway II describes the joint contribution of the genetically heterogeneous *α_p_*, *α_s_*, and *α_eut_* constituents. Accordingly, the observed correlation between an increasing amount of the secondary structural constituent and decreasing shrinkage does not demonstrate a direct causal effect of the γ_2_ phase. The quantities *φ_D_* and *φ_γ_*_2_ are more appropriately regarded as structural indicators of a change in the temperature–phase state.

The bronzes assigned to pathways II and III at *K_R_* = 0.46 and *K_R_* = 0.45 have the same reported experimental final shrinkage of 1.41%. The difference of 0.0026 percentage points between their calculated shrinkage values is not established as experimentally significant, as discussed in Section 4.2. The nearly compensating changes in the calculated thermal and structural-transformation terms describe a model-dependent allocation of the deformation balance. They are conditional on the reconstructed pathways, parameterization, and transfer assumption and do not independently demonstrate an abrupt physical change between these neighboring compositions. In particular, the separate component magnitudes and their compensation were not measured experimentally.

A change in the genetic pathway is also accompanied by a redistribution of the calculated deformation balance among the successive temperature intervals. Under the adopted comparative representation, the combined relative contribution assigned to ΔT_1_ and ΔT_2_ increases from 47–49% for pathway I to 64–67% for pathway II and 80–83% for pathway III. The integral structural-transformation contribution, formally included in the resultant ΔT_3_ contribution for balance closure, cannot be physically localized within a specific temperature interval on the basis of the available experimental data. Thus, when the initial state changes to *β_p_*, a larger portion of the final deformation balance in the model shifts to the high-temperature region.

The present model reconstructs integral free shrinkage under the investigated experimental conditions. It does not calculate casting–mold restraint, spatial temperature gradients, stress evolution, or the resulting distortion and cracking. Application to constrained castings would require a coupled thermal–mechanical formulation with appropriate geometry, boundary and contact conditions, and temperature-dependent mechanical properties, including inelastic deformation and stress relaxation where relevant. Following independent validation, the reconstructed contraction characteristics could be considered as candidate inputs to such a formulation; they do not by themselves provide predictions of residual stresses or casting distortion. These applications remain a direction for future research.

The reported inverse-model accuracy characterizes fitting to the identification dataset, rather than prediction of independent compositions. The linear benchmark already achieves a leave-one-composition-out RMSE of 0.00915 percentage points. Additional predictive value of the inverse model over this benchmark has therefore not been demonstrated. Its contribution in the present study is a conditional framework for examining how measured thermal and structural descriptors can be represented within a calculated deformation balance. This interpretive contribution remains subject to the limitations of the reconstructed pathways, parameter transfer, and parameter identifiability discussed above.

The quantitative results in Section 4.4 show that pathway III is sensitive to the limited experimental sample. Its normalized condition number is 67.88, omission of the *K_R_* = 0.45 composition substantially changes both fitted parameters, and the newly computed conditional pathway-III bootstrap interval for k_III_ crosses zero. The negative baseline estimate and its persistence in the transferred-coefficient sensitivity scenarios should therefore not be generalized to sign stability under resampling of these five compositions. This bootstrap result is conditional on fixed upstream characteristics and does not quantify uncertainty throughout the cascade.

Table 10 demonstrates functional-form sensitivity even when the upstream reconstruction is held fixed: similar total-shrinkage fits coexist with substantially different thermal and structural-transformation allocations. Consequently, the compensating changes reported for the baseline linear model should not be interpreted as a parameterization-independent physical mechanism.

Independent physical validation would require additional observables that constrain the two contributions separately. Temperature-resolved dilatometry under comparable cooling conditions, combined with in situ measurements of phase fractions and lattice parameters, could provide such constraints and help distinguish thermal contraction from transformation-related dimensional changes. These measurements were not available in the present study. Consequently, the reported component magnitudes and their compensating changes are treated as model-dependent estimates rather than experimentally verified individual strain contributions.

Thus, the principal model-based interpretation proposed in this study is a structure-dependent change in the calculated temperature–deformation balance within the adopted genetic pathways:

*K_R_* → genetic pathway → structural states → IA-CLTCs and *k_p_* → deformation balance → *α_f_*.

Related methodological challenges have been discussed for electrocaloric materials, where differences in composition, processing conditions, and measurement protocols complicate comparisons between datasets and the evaluation of predictive models [47].

## 6. Conclusions

Three genetic structure-formation pathways were proposed for CuAl7Si2Sn1.5Mn0.3 bronze over *K_R_* = 0.41–0.85 through joint interpretation of thermal analysis and metallographic observations, with the Cu–Al phase diagram used as a qualitative reference. The pathways provide structural schemes for the inverse model; their intermediate phase assignments, particularly the inferred change between pathways II and III, require independent verification.

The methodological contribution is an inverse framework that links integral free shrinkage to effective interval-averaged characteristics of genetically successive structural states within adopted reconstruction schemes. Its distinction from conventional homogenization is the identification of these effective state characteristics without a complete set of intrinsic phase properties. The least-squares solution is an established estimation tool; the contribution concerns the structural parameterization and cascade organization of the inverse problem. The identified coefficients remain conditional model parameters, and their estimation does not independently establish the uniqueness of the reconstructed pathways.

Within the adopted inverse model, the smooth experimental *α_f_* = f(*K_R_*) relationship is compatible with substantial changes in the calculated component balance. Near the boundary between pathways II and III, the model assigns nearly compensating changes to the thermal and structural-transformation terms; these changes have not been independently verified experimentally.

Within the adopted parameterization, the calculated thermal contributions shift toward the upper-temperature intervals from pathway I to pathway III. This redistribution is model-dependent. The available experimental data do not determine the temperature localization of the integral structural-transformation contribution.

The inverse model fitted the shrinkage of the 17 investigated bronzes with *R*^2^ = 0.998 and RMSE = 0.00562 percentage points. These are identification-dataset metrics. The simpler linear regression achieved a leave-one-composition-out RMSE of 0.00915 percentage points; superior predictive performance of the inverse model has not been established. The proposed approach is therefore presented as a conditional reconstruction framework rather than a validated improvement in shrinkage prediction.

The thermal and structural-transformation contributions were inferred jointly from integral shrinkage data and were not measured independently. Their magnitudes and compensating behavior should therefore be regarded as model-dependent estimates within the investigated compositions and cooling conditions. Numerical stability and agreement with total shrinkage do not establish a unique physical decomposition; independent validation is required before the individual contributions are used as experimentally established inputs for further thermomechanical modeling.

## Figures and Tables

**Table 1:** Nominal composition range [26] and experimentally determined chemical compositions of the investigated CuAl7Si2Sn1.5Mn0.3 bronzes with different *K_R_* values.

Nominal Composition Range (wt.%)		Al	Si	Mn	Sn	Cu + Impurities
CuAl7Si2Sn1.5Mn0.3		6.0–7.5	1.0–2.5	0.21–0.45	1.0–2.2	Balance
*K_R_*	Mass fractions of chemical elements (wt.%)					Total impurities, nn,%
	Cu	Al	Si	Mn	Sn	
0.85	89.715	6.65	1.58	0.438	1.33	0.287
0.84	91.036	6.12	1.02	0.212	1.41	0.202
0.80	89.601	6.45	1.88	0.282	1.31	0.477
0.77	88.777	7.07	2.09	0.322	1.45	0.291
0.76	89.006	6.88	1.98	0.358	1.44	0.336
0.73	89.733	6.02	2.44	0.289	1.12	0.398
0.68	89.775	6.23	1.98	0.262	1.42	0.333
0.63	89.827	6.22	1.72	0.388	1.55	0.295
0.59	87.928	7.49	1.89	0.297	1.99	0.405
0.55	88.418	6.73	2.41	0.448	1.65	0.344
0.50	87.981	7.09	2.24	0.395	1.99	0.304
0.46	87.089	7.47	2.42	0.437	2.16	0.424
0.45	88.16	6.96	2.01	0.392	2.18	0.298
0.44	88.607	6.61	2.06	0.274	2.11	0.339
0.43	88.475	6.71	2.13	0.229	2.18	0.276
0.42	87.687	7.07	2.4	0.355	2.2	0.288
0.41	87.662	6.97	2.33	0.437	2.19	0.411

Note: The upper part of the table specifies the adopted nominal composition range of CuAl7Si2Sn1.5Mn0.3 bronze, whereas the lower part presents the experimentally determined chemical compositions of the 17 investigated bronzes. The symbol nn denotes the total impurity content (wt.%). The composition criterion *K_R_* was calculated using Equation (1) and is reported to two decimal places.

**Table 2:** Genetic temperature–phase pathways of structure formation in the investigated bronzes.

Pathway	*K_R_* Range	Temperature Interval of Structural-State Existence		
		1 (Δ*T*_1_ = *T_S_* − *T_SU_*)	2 (Δ*T*_2_ = *T_SU_* − *T_eut_*)	3 (Δ*T*_3_ = *T_eut_* − *T*_0_)
I	0.71 < *K_R_* ≤ 0.85	*α_p_*	*α_p_* + *β_s_*	*α_p_* + *α_eut_* + *γ*_2_
II	0.45 < *K_R_* ≤ 0.71	*α_p_* + *β_p_*	*α_p_* + *α_S_* + *β_p_*	*α_p_* + *α_S_* + *α_eut_* + *γ*_2_
III	0.41 ≤ *K_R_* ≤ 0.45	*β_p_*	*α_S_* + *β_p_*	*α_S_* + *α_eut_* + *γ*_2_

Notes: The subscript *p* denotes the primary origin of a structural constituent formed during solidification; *s* denotes the secondary origin of a constituent formed by a solid-state transformation; and *eut* denotes formation of the *α_eut_* constituent during the final eutectoid transformation. *T_S_*, *T_SU_*, and *T_eut_* are the solidus, solvus, and eutectoid-transformation temperatures, respectively; *T*_0_ is room temperature. The following relation holds: Δ*T*_1*,i*_ + Δ*T*_2*,i*_ + Δ*T*_3*,i*_ = *T_S,i_* − *T*_0_.

**Table 3:** Numbers of specimens and half-ranges of experimental free linear shrinkage for the investigated bronze compositions.

Kᵣ	Number of Specimens, n	Half-Range of Measured Shrinkage, Percentage Points	Kᵣ	Number of Specimens, n	Half-Range of Measured Shrinkage, Percentage Points
0.85	7	0.0043	0.55	4	0.0037
0.84	4	0.0043	0.50	7	0.0034
0.80	7	0.0042	0.46	5	0.0032
0.77	7	0.0038	0.45	4	0.0030
0.76	5	0.0037	0.44	4	0.0029
0.73	4	0.0038	0.43	6	0.0029
0.68	5	0.0033	0.42	4	0.0031
0.63	6	0.0035	0.41	4	0.0028
0.59	4	0.0031			

**Table 4:** Experimental parameters of structure formation and free linear shrinkage in the investigated bronzes.

Pathway	*K_R_*	T_S_, °C	T_SU_, °C	Tₑᵤₜ, °C	$\varphi_{D}$, %	$\alpha_{f}^{exp}\equiv \alpha_{B}$, %
I	0.85	971.50	541.80	540.80	11.5	1.77
	0.84	970.00	555.56	541.70	13.3	1.76
	0.80	972.20	609.20	543.90	14.4	1.74
	0.77	974.40	649.43	544.20	14.9	1.71
	0.76	975.60	662.50	549.30	17.5	1.68
	0.73	974.70	724.00	552.30	20.2	1.65
II	0.68	976.10	700.00	553.30	26.9	1.60
	0.63	973.90	677.00	549.90	34.9	1.56
	0.59	975.30	659.90	551.40	35.8	1.54
	0.55	971.50	648.50	548.90	43.2	1.50
	0.50	966.70	627.20	548.60	44.3	1.46
	0.46	963.40	611.20	551.20	53.1	1.41
III	0.45	964.00	608.10	549.50	54.8	1.41
	0.44	964.40	607.90	547.20	56.4	1.39
	0.43	962.70	606.70	548.10	56.9	1.39
	0.42	961.10	605.10	548.40	57.2	1.38
	0.41	960.40	605.00	546.30	58.1	1.37

Note: The parameter *φ_D_* is the experimentally determined area fraction of the final dark eutectoid structural constituent (*α_eut_* + γ_2_) after the bronze has cooled to room temperature. Under the adopted stereological approximation, the area fraction *φ_D_* was used as an estimate of the volume fraction of this structural constituent.

**Table 5:** Experimental metallographic fractions, predictions of Equation (14), and derived constituent fractions for the investigated bronzes.

*K_R_*	$\varphi_{D}^{exp},\%$	$q_{\gamma_{2}}^{exp},\%$	$q_{\gamma_{2}}^{calc},\%$	$\varphi_{\alpha ,eut},\%$	$\varphi_{\gamma_{2}},\%$
0.85	11.5	93.80	93.8	0.7	10.8
0.84	13.3	92.20	92.5	1.0	12.3
0.80	14.4	88.00	87.6	1.7	12.7
0.77	14.9	84.30	84.1	2.3	12.6
0.76	17.5	81.80	83.0	3.2	14.3
0.73	20.2	80.30	79.8	4.0	16.2
0.68	26.9	74.20	75.0	6.9	20.0
0.63	34.9	70.20	70.9	10.4	24.5
0.59	35.8	69.10	68.0	11.1	24.7
0.55	43.2	64.70	65.5	15.2	28.0
0.50	44.3	62.40	62.9	16.7	27.6
0.46	53.1	63.70	61.3	19.3	33.8
0.45	54.8	60.80	60.9	21.5	33.3
0.44	56.4	60.30	60.6	22.4	34.0
0.43	56.9	61.20	60.3	22.1	34.8
0.42	57.2	59.80	60.1	23.0	34.2
0.41	58.1	58.30	59.8	24.2	33.9

Note: φ_D,exp_ is the mean area fraction of the dark eutectoid region. q_γ2,exp_ is the experimentally estimated relative area fraction of the constituent assigned as γ_2_ within that region. q_γ2,calc_ was calculated using Equation (14) and is displayed as a percentage. The last two columns are stereological volume-fraction estimates derived from the experimental area fractions. Calculations used unrounded intermediate values; displayed values are rounded. Composition-specific standard deviations and confidence intervals are not available.

**Table 6:** Effective IA-CLTCs, their identification sources, and available sensitivity information.

Structural Constituent or State	IA-CLTC, 10^−6^ K^−1^	Physical Interpretation and Identification Source
*α_p_*	20.10	Fixed reference IA-CLTC of the initial primary α state in pathway I, adopted before cascade identification.
*αᴵ*	19.97	Effective combined α characteristic obtained by auxiliary decomposition of the final structural state of pathway I.
*αᴵᴵ*	11.24	Effective combined α characteristic obtained by auxiliary decomposition of the final structural state of pathway II.
*γ*_2_	12.50	Effective parameter obtained by joint auxiliary decomposition of the final structural states of pathways I and II.
*β_p_*	25.90	Effective initial-state characteristic identified by least squares for pathway III, conditional on the transferred upstream characteristics.
*α_S_ + β_p_*	36.654	Effective β-derived subsystem characteristic extracted from pathway II and transferred to pathway III as a closure assumption.
*α_S_ + α_eut_ +* *γ*_2_	11.66–11.68	Composition-dependent effective characteristic reconstructed for the final state of pathway III

Note: IA-CLTCs are expressed in 10^−6^ K^−1^ and represent effective model characteristics. The αp value is a fixed reference, not an independently estimated parameter of the subsequent pathway-I fit. Conditional sensitivity ranges for the auxiliary constituent estimates are reported below. The range 11.66–11.68 describes variation among compositions, not a statistical uncertainty interval.

**Table 7:** Full-data fitting and cross-validation results for the linear benchmark, compared with the reported full-data fit of the inverse model.

Model	Evaluation	*R*^2^/*Q*^2^	RMSE, Percentage Points	MAPE, %
Linear regression, Equation (16)	Full-data fit	0.99671	0.00818	0.419
Linear regression, Equation (16)	Leave-one-composition-out cross-validation	0.99589	0.00915	0.467
Inverse temperature–phase model	Full-data fit, reported results	0.998	0.00562	0.266

Note: R^2^ denotes the coefficient of determination for full-data fitting, whereas Q^2^ denotes the cross-validated coefficient of determination calculated from pooled leave-one-composition-out predictions. RMSE and MAPE are the root mean square error and mean absolute percentage error, respectively. The linear-regression results were calculated using the rounded experimental data in Table 4. The inverse temperature–phase model results represent full-data fitting to the 17 compositions used for parameter identification. Conditional cascade leave-one-composition-out cross-validation was also performed for the inverse model, with the reference IA-CLTC of the primary α_p_ state fixed at 20.10 × 10^−6^ K^−1^. Therefore, the full-data fitting and cross-validation metrics characterize different aspects of model performance and should not be interpreted as directly equivalent.

**Table 8:** Conditional sensitivity of pathway-III parameter estimates and calculated shrinkage to variation in the transferred IA-CLTC.

Variation, %	Transferred IA-CLTC	Change in *β_p_* IA-CLTC	Change in k_III_	Maximum Absolute Shrinkage Change
−20	29.3232	+1.448958	−0.000381521	0.001315269
−10	32.9886	+0.724479	−0.000190761	0.000657634
−5	34.8213	+0.362240	−0.000095380	0.000328817
0	36.6540	0	0	0
+5	38.4867	−0.362240	+0.000095380	0.000328817
+10	40.3194	−0.724479	+0.000190761	0.000657634
+20	43.9848	−1.448958	+0.000381521	0.001315269

Note: Changes are relative to the baseline least-squares solution. Results are approximate because the published input data are rounded. The final column describes changes from the baseline calculated shrinkage, not errors relative to experimental measurements. All inputs other than the transferred coefficient and the two re-estimated parameters were held fixed.

**Table 9:** Previously reported cascade Monte Carlo intervals and newly computed conditional pathway-III bootstrap intervals.

Parameter	Cascade Monte Carlo: 2.5th–97.5th Percentiles	Conditional Pathway-III Bootstrap: 2.5th–97.5th Percentiles
IA-CLTC of the initial *β_p_* state, 10^−6^ K^−1^	19.995 to 31.804	10.3843 to 30.5281
k_III_, percentage points per vol.%	−0.023434 to −0.008833	−0.023216 to 0.008323

Note: The columns represent different computational procedures and scopes. The Monte Carlo column retains the previously reported cascade results and was not recomputed in this revision. Its perturbation scales were based on fitting residuals, and uncertainties in transformation temperatures and metallographic fractions were not jointly propagated. The bootstrap column reports a new exhaustive resampling analysis of the five pathway-III compositions with upstream characteristics fixed. Its intervals are conditional on the 3120 retained full-rank resamples; the complete calculation script and acceptance log are provided in Supplementary File S1. The interval widths should not be compared as estimates of the same uncertainty. Neither procedure provides a complete experimental measurement-uncertainty budget.

**Table 10:** Conditional sensitivity of pathway-III identification and shrinkage decomposition to the structural-transformation functional form.

Quantity	Linear	Constrained Quadratic
*β_p_* IA-CLTC, 10^−6^ K^−1^	25.9042	16.1963
k_III_, percentage points per vol.%	−0.0161464	+0.0151328
c_III_, percentage points per (vol.%)^2^	—	−0.000707026
Fitting RMSE, percentage points	0.005532	0.005442
Thermal contribution at *K_R_* = 0.45, %	1.751830	1.406324
Structural-transformation contribution at *K_R_* = 0.45, percentage points	−0.345589	0
Calculated total shrinkage at *K_R_* = 0.45, %	1.406240	1.406324

Note: Both fits use the same five compositions and fixed upstream characteristics. Values were calculated from rounded published inputs. The quadratic structural-transformation term reaches its upper bound of zero at *K_R_* = 0.45. A positive quadratic-model k_III_ does not imply a positive total structural-transformation contribution because the quadratic term must also be included. These are conditional fitting results, not cross-validation errors.

**Table 11:** Comparison of the predictive performance of linear and quadratic structural-transformation parameterizations using conditional cascade leave-one-composition-out cross-validation (LOOCV).

Parameterization	Pathway I RMSE	Pathway II RMSE	Pathway III RMSE/Overall RMSE
Linear	0.012779	0.006828	0.009213/0.009953
Unconstrained quadratic	0.033399	0.010044	0.045690/0.032328
Fully constrained quadratic	0.024164	0.007044	0.015016/0.017027

Note: All RMSE values are expressed in percentage points of free linear shrinkage. The reported results correspond to conditional cascade LOOCV with the reference IA-CLTC of the primary α_p_ state fixed at 20.10 × 10^−6^ K^−1^. The uncertainty associated with the initial determination of this reference coefficient was not included.

## Data Availability

The original contributions presented in this study are included in the article/Supplementary Material. Further inquiries can be directed to the corresponding authors.

## Supplementary Materials

The following supporting information can be downloaded at: https://www.mdpi.com/article/10.3390/ma19214622/s1. Supplementary File S1: Python calculation script and complete acceptance log for the exhaustive conditional pairs-bootstrap analysis of pathway III (referenced in Section 3.5).
